# Transforming labor requirement, crop yield, and profitability with precision dry-direct seeding of rice and integrated weed management in Eastern India

**DOI:** 10.1016/j.fcr.2020.107961

**Published:** 2020-12-15

**Authors:** P. Panneerselvam, Virender Kumar, Narayan Chandra Banik, Vivek Kumar, Nabakishore Parida, Iftikar Wasim, Aurovinda Das, Sanghamitra Pattnaik, Pravat Kumar Roul, Dilip Ranjan Sarangi, Pardeep K. Sagwal, Peter Craufurd, Balwinder-Singh, Ashok Yadav, Ram K. Malik, Sudhanshu Singh, Andrew J. McDonald

**Affiliations:** a International Maize and Wheat Improvement Centre, NASC Complex, New Delhi, India; b International Rice Research Institute, Los Baños, Philippines; c International Rice Research Institute, NASC Complex, New Delhi, India; d Odisha University of Agriculture and Technology, Bhubaneshwar, India; e National Rice Research Institute, Cuttack, India; f International Maize and Wheat Improvement Centre, Kathmandu, Nepal; g Soil and Crop Sciences Section, School of Integrative Plant Sciences, Cornell University, Ithaca, NY, USA

**Keywords:** fb, followed by, DSR, direct seeded rice, IWM, integrated weed management, DAS, days after sowing, PRE, pre-emergence herbicide, POST, post-emergence herbicides, HW, hand weeding, MW, mechanical weeder, SR, seed rate, Dry-direct seeding, Drill-DSR, Precision broadcast-DSR, On-farm research, Beushening, Herbicide

## Abstract

In many parts of Eastern India that have a very high prevalence of rural poverty and food insecurity, the prevailing rice establishment practice of ‘*beushening*’ is characterized by low yields and modest profitability, while labor and energy inputs are high. *Beushening* consists of broadcasting ungerminated rice seed at high rates (>100 kg ha^−1^) prior to the onset of monsoon rain, followed by ploughing at 4–6 weeks after crop emergence to control weeds with subsequent manual gap filling through seedling redistribution to ensure stand uniformity. Dry-direct seeding of rice (DSR), both drill-DSR and precision broadcast-DSR in combination with integrated weed management (IWM) may offer a pathway for simultaneously reducing costs and markedly increasing productivity. On-farm trials were conducted from 2016 to 2018 in four districts of Odisha (Mayurbhanj, Cuttack, Bhadrak, and Puri) to evaluate the yield and economic performance of dry-DSR (drill and precision broadcast), coupled with herbicide-based IWM strategies, in comparison with conventional *beushening*. Drill-DSR with IWM increased grain yield by 1.7 t ha^−1^ in Mayurbhanj and 1.3 t ha^−1^ in Cuttack, but not in Bhadrak, compared to *beushening*. The combination of increased yield and lower variable cost in drill-DSR increased net benefit by 550, 395, and 166 US$ ha^−1^ in Mayurbhanj, Cuttack, and Bhadrak, respectively. For farmers without access to seed drills, precision broadcast-DSR with IWM increased yields by 0.91, 1.22 and 0.60 t ha^−1^, and net benefits by 270, 312, and 188 US$ ha^−1^ in Mayurbhanj, Puri, and Bhadrak, respectively. Among the IWM practices evaluated in dry-DSR, application of pretilachlor + safener @ 500 g *ai* ha^−1^ as pre-emergence, followed by bispyribac-sodium @ 20 g *ai* ha^−1^ at 15–25 days after sowing as post-emergence, and then one spot hand weeding at 30–35 days after sowing was effective in controlling weeds. These results suggest that rice yield gaps in eastern India can be reduced, and farmers’ income from rice can be increased by more than 50 % by replacing *beushening* with drill-DSR or precision broadcast-DSR. The results could be applicable to approximately 6.4 million ha of lowland rice where *beushening* is currently practiced in Eastern India.

## Highlights

• Dry seeded rice (DSR) [drill and precision broadcast] was tested over *beushening*.
• Dry Drill-DSR resulted in US$ 166−550 ha^−1^ higher net benefit than *beushening*.
• Net benefit gain from precision broadcast-DSR was US$ 188−312 ha^−1^ over *beushening*.
• Effective integrated weed management options were identified for DSR.
• Other benefits of DSR were reduction in seed rate, labor and production cost.

## 1 Introduction

Rice is one of the most important cereals for global food security, especially in Asia, where it is a staple food for about half of the world population (GRiSP, 2013). India is the world’s top rice producing country in terms of area and ranked second in terms of production. About 25 % of world’s rice is grown in India, contributing to 21 % of global rice production (FAO, 2018). To meet global rice demand, it is projected that an additional 96 million tons of milled rice will be needed by 2040 as compared to 2015 (Valera and Balié, 2020). The challenge is that this additional rice must be produced with a lower environmental footprint (using less water, labor, agro-chemicals) in changing climates to ensure both food security and environmental sustainability. One approach to achieve this target is by closing rice yield gaps, especially in areas such as the Eastern States of India where these yield gaps are large.

In Eastern India, rice is grown on 24.6 million ha, primarily rainfed, and rice is the most common crop during *kharif* (monsoon) season when heavy rainfall and inundated soil conditions favour its cultivation (Adhya et al., 2008; Kumar et al., 2010). Average rice yield in this region is low as compared to national average because of (i) in-season monsoon variability, despite high total rainfall, and (ii) limited use of best agronomic practices (Panneerselvam and Sudhanshu, 2019). For example, 6.4–10.2 million ha of rainfed lowland rice in the region is cultivated with a traditional method known as *beushening* (Nayak and Lenka, 1988; Tomar, 2002; Das, 2012; Gautam et al., 2013; Pandey et al., 2018). Locally, this practice is known as beushen in Odisha and Bihar, biasi in eastern Madhya Pradesh and Chhattisgarh, lev in Eastern Uttar Pradesh, and baug or bidauni in Bihar (Tomar, 2002).

The *beushening* method consists of broadcasting ungerminated rice seeds using high seed rates (>100 kg ha^−1^) in the field before the onset of monsoon rain, followed by cross-ploughing and laddering (leveling using flat wooden plank) at 4–6 weeks after emergence when 10−15 cm of rainwater has accumulated in rice fields. Cross-ploughing and laddering helps to control weeds, thins the crop stand, and distributes rice seedlings more evenly (Singh et al., 1994; Tomar, 2002). These operations are labor-intensive, tedious, and are largely carried out by women. However, sub-optimal crop management (e.g. no early weed control or fertilizer application prior to *beushening* operation) and rainfall-dependent *beushening* operations lead to low yield (Tomar, 2002). Nonetheless, *beushening* is widely practiced by resource poor farmers in Eastern India because they obtain stable low yields under a highly variable climate from limited investment in inputs (Nayak and Lenka, 1988).

A high variable cost associated with high seed rate and labor, together with low yield, is common to *beushening* and reduces profitability. In areas that are mechanizing, dry-direct seeding (dry-DSR) established with seed drills (drill-DSR) and coupled with integrated weed management (IWM) may provide an alternative to *beushening*. Drill-DSR helps achieve regular crop geometry and optimum plant density, making it easier for intercultural operations for weed (manual or mechanical), pest and disease, irrigation, and fertilizer management. Drill-DSR has the potential to reduce the variable cost by reducing labor and energy requirements compared to *beushening* (CSISA, 2017). Wherever seed drills are not available, another dry-DSR method, namely precision broadcast-DSR (i.e. careful manual precision broadcasting of seed at a lower rate), combined with IWM could be an important alternative. Precision broadcast-DSR reduces labor use, thereby reducing drudgery to women compared to *beushening*.

Recently, there is also increased interest to shift from puddled transplanted rice (PTR) – the most dominant rice cultivation method in Asia to direct-seeded rice (DSR) to address major drivers of agricultural change in the region. These drivers include labor and water scarcity, as well as rising costs of cultivation (Kumar and Ladha, 2011; Chakraborty et al., 2017; Hellin et al., 2020). DSR can be established by three methods: dry-seeding (Dry-DSR), wet-seeding (wet-DSR), and water seeding (Kumar and Ladha, 2011; Rao et al., 2017). In dry-DSR, rice seeds (not germinated, but can be primed) are sown in non-puddled soil, whereas in wet-DSR, pre-germinated (sprouted) seeds are sown on puddled/wet soil. In water-seeding, pre-germinated seeds are sown in standing water on a puddled or non-puddled soil. In areas where labor scarcity is the major constraint, but water is readily available or cheap, farmers are shifting from PTR to wet-DSR. However, in areas where both water and labor are becoming increasingly scarce, as is the case in South Asia, dry-DSR is preferred (Pandey and Velasco, 2005; Rao et al., 2017) and the focus is on development and deployment of dry-DSR (Kumar and Ladha, 2011; Laik et al., 2014; Kumar et al., 2018; Kakumanu et al., 2019). Dry-DSR in South Asia has shown potential to improve economic sustainability and reduce the environmental footprint of rice cultivation as it: (i) saves labor, water and energy, and hence reduces cost of cultivation and increases net income; and (ii) reduces greenhouse gas (GHG) emissions (Kumar and Ladha, 2011; Gathala et al., 2013; Ladha et al., 2016; Padre et al., 2016; Chakraborty et al., 2017; Kumar et al., 2018; Kakumanu et al., 2019). Because of above mentioned benefits, farmers are slowly transitioning from PTR to dry-DSR in various parts of South Asia (CSISA, 2017; Bhullar et al., 2018; Devkota et al., 2019; Kakumanu et al., 2019).

Weeds are considered as one of the major constraints to wide-scale adoption of dry-DSR (Rao et al., 2007; Kumar and Ladha, 2011; Kumar et al., 2013; Rao et al., 2017; Xu et al., 2019). The weeds and the crop emerge at the same time with weeds growing more quickly (Rao et al., 2007 and 2017), leading to 50–90 % yield reduction if weeds are not properly controlled (Chauhan and Johnson, 2011; Singh et al., 2005). When weeds are effectively controlled, DSR yields are similar to that of transplanted rice (Ali et al., 2006; Gathala et al., 2013; Kukal and Aggarwal, 2002). In Eastern India, in both *beushening* and in PTR, weeds are controlled mainly by hand-weeding. Manual hand-weeding is becoming difficult and uneconomical due to labor scarcity at the critical time of weeding (Chauhan, 2012; CSISA, 2017; Kumar and Ladha, 2011). Hence, alternative crop establishment methods and effective herbicide based-integrated weed management (IWM) practices are needed to reduce variable costs and labor use/cost.

There is a knowledge gap on the performance of drill-DSR seeding as compared to *beushening*. There is also a need to identify cost-effective IWM options for weed control in drill-DSR and precision broadcast-DSR in rainfed lowland environments. We hypothesized that both drill-DSR and precision broadcast-DSR with IWM will result in higher net benefits compared to *beushening*, due to a combination of lower variable cost and higher yield. To test the above hypothesis, three types of experiments were conducted in farmers’ fields from 2016 to 2018 with the following objectives: (1) to evaluate the performance of drill-DSR as compared to *beushening*; (2) to identify effective and profitable herbicide-based IWM options for drill-DSR; and (3) to evaluate the combination of lower seed rate and IWM options for precision broadcast-DSR as an alternative to *beushening* for those areas with limited access to seed-drills.

## 2 Materials and methods

### 2.1 Study area

**Fig. 1:**
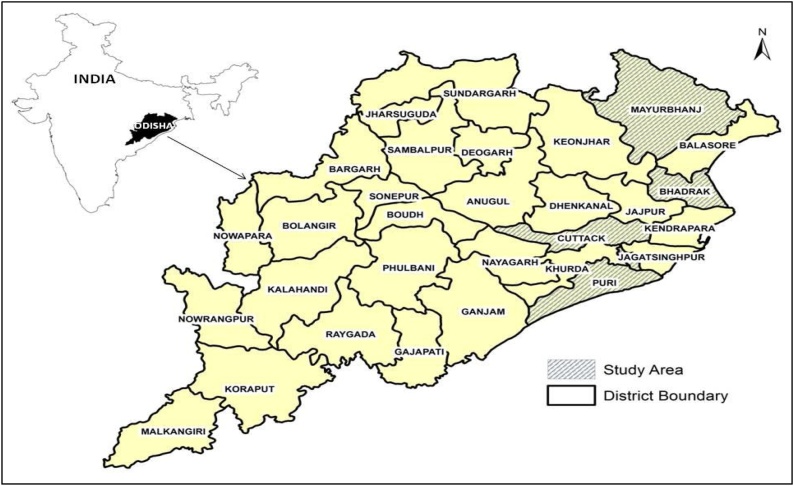
Map for Odisha state in India showing four districts of study area.

Experiments were conducted in farmers’ fields in the kharif or monsoon season across three years (2016–2018) in four districts of Odisha (Fig. 1), including Bhadrak (21.0126 °N, 86.6208 °E), Cuttack (20.5168 °N, 85.7256 °E), Mayurbhanj (22.0087 °N, 86.4187 °E), and Puri (19.8510 °N, 85.7256 °E). The climate and other site characteristics of four studied districts are presented in Table 1. The experiments were laid out in following villages: Narayan pur, Neulia, Kandagadia, Khirosahi, Todanga, Adia, and Odang in Bhadrak; Agria, Sikarghati, Belpal, Bada brahmanamora, Chilbasa, Chhuruni, Bishnurpur, Renugaon, Kansapal, and Pratappu in Mayurbhanj; Haridapal in Cuttack; and Danogahir, Salajangha, Resinga and Maniancha in Puri.

**Table 1:** Climate, soil and other characteristics of four studied districts in Odisha (Das, 2012).

	Mayurbhanj	Bhadrak	Puri	Cuttack
Agro-climatic zone	North central plateau	North Eastern Coastal Plain Zone	East and southeastern coastal plain	East and southeastern coastal plain
Climate	Sub-tropical – hot and moist	Sub-tropical – hot and humid	Sub-tropical – hot and humid	Sub-tropical – hot and humid
Annual rainfall (mm)	1648	1568	1450	1577
Monsoon rainfall (mm)	1361	1376	1087	1467
Major cropping systems	Rice- fallow, Rice-pulses/oilseeds, Maize-fallow	Rice-pulses/oilseeds, Rice- fallow	Rice-pulses/oilseeds, Rice-Rice, Rice-fallow	Rice-pulses/oilseeds, Rice- fallow
Cropping intensity (%)	121	138	207	153
Kharif rice area (ha)	339,000	165,000	119,000	128,000
Highland (%)	26	2	4	8
Medium Land (%)	37	38	25	40
Lowland (%)	37	60	71	52
Irrigated rice area in kharif season (%)	28	62	72	68
Major risk and uncertainties	Intermittent drought, lack of life saving irrigation	Flood, cyclone, saline soil, submergence in lowland rice	Flood, cyclone and submergence in lowland rice	Flood, cyclone and submergence in lowland rice
Soil type	Laterite and red soil	Red and laterite, deltaic alluvium, coastal saline	Coastal saline, sandy, lateritic, alluvial, black and red	Coastal saline, sandy, lateritic, alluvial, black and red
Soil texture	Sandy loam	Loam and clay loam	Coastal alluvial saline loamy sand to clay loam	Coastal alluvial saline

### 2.2 Experimental details

#### 2.2.1 Experiment I: assessing performance of drill-DSR compared to bueshening

This experiment was conducted in Bhadrak, Mayurbhaj and Cuttack in 2017 and 2018 under rainfed lowland conditions (N = 24). Two establishment methods, viz *beushening* and drill-DSR, were tested at five farmers’ fields for each year in Bhadrak and Mayurbhanj, and at four farmers’ fields in Cuttack in 2018. The treatments were not replicated at each farmer’s field, and farmers’ fields were used as replications. The size of the experimental plots was 200 m^2^ in Bhadrak, 800−1000 m^2^ in Mayurbhanj, and 400 m^2^ in Cuttack. The experimental area for both treatments was cross-tilled by four-wheel tractor with cultivator to a depth of about 15 cm prior to sowing. One light planking was used after sowing in both treatments to crush the hard clods and smooth/compact the soil lightly to ensure that seeds were covered with soil. This helps to avoid any phytotoxicity from the pre-emergence herbicide, ensures better seed germination, and avoids moisture loss just after sowing.

In the *beushening* treatment, weeds were managed by *beushening* operation, which includes recurrent tillage and laddering followed by hand weeding and redistribution of seedlings. In drill-DSR, weeds were managed either by the application of pretilachlor with safener (Sofit/Eraze-N) @ 500 g *ai* ha^−1^ as pre-emergence (PRE) followed by hand-weeding at 20–25 days after sowing (DAS) or by post-emergence (POST) application of tank mix of bispyribac-sodium + pyrazosulfuron- ethyl @ 20 + 20 g *ai* ha^−1^ at 15–25 DAS followed by one hand-weeding at 30–35 DAS. In Mayurbhanj, Swarna (MTU 7029) variety was sown in drill-DSR @ 45 kg ha^−1^ using seed-cum-fertilizer drill, and by broadcast method using a seed rate of 100 kg ha^-1^ in *beushening*. In Bhadrak and Cuttack, Swarna Sub-1 at the seed rate of 45 kg ha^−1^ was sown using seed-cum-fertilizer drill in drill-DSR, and by broadcast method with seed rate of 100 kg ha^−1^ in *beushening*. Fertilizer rates were similar in both drill-DSR and *beushening* treatments at each farmers’ field but varied across districts (Table 2). In *beushening*, full P and K in the form of diammonium phosphate (DAP) and muriate of potash (MOP), respectively and 50 % N in the form of urea and DAP were broadcast immediately after *beushening* operation (30–40 DAS). The remaining N in the form of urea was broadcast at panicle initiation. In drill-DSR, full P, K and 33 %N were applied at the time of sowing in the form of DAP, MOP, and urea, and remaining N in the form of urea was broadcast in two equal splits at 25–30 DAS and at panicle initiation. Pest and disease management in all experiments followed farmers’ practices.

**Table 2:** Fertilizer rates (kg ha^−1^) in Experiments I, II, and III.

	N	P_2_O_5_	K_2_O
Experiment I*			
Bhadrak	81	61	45
Mayurbhanj	91	57	45
Cuttack	126	57	37
Experiment II*			
Bhadrak	106	48	37
Mayurbhanj	92	57	45
Experiment III*			
Bhadrak	68	59	40
Mayurbhanj	71	35	37
Puri	61	30	41

* Within each district, the fertilizer rate was fixed and the same for all treatments in each year but varied across districts.

#### 2.2.2 Experiment II: optimizing weed management in drill-DSR

This experiment was conducted at farmers’ fields in Bhadrak and Mayurbhanj districts in 2017 and 2018 under rainfed lowland conditions (N = 20). Treatments included:

T1: Hand weeding twice, at 15–20 DAS and 30–35 DAS;

T2: Pretilachlor with safener (Sofit/Eraze-N) @ 500 g *ai* ha^−1^ PRE followed by (*fb)* hand weeding at 20–25 DAS;

T3: Tank mix of bispyribac -sodium + pyrazosulfuron -ethyl @ 20 + 20 g *ai* ha^−1^ at 15–25 DAS as POST;

T4: Tank mix of bispyribac- sodium + pyrazosulfuron- ethyl @ 20 + 20 g *ai* ha^−1^ at 15–25 DAS as POST *fb* one hand weeding at 30–35 DAS;

T5: One mechanical weeding using paddy power weeder at 15–20 DAS.

Each treatment was replicated at five farmers’ fields in each district for both years. The size of the experimental plot was 400 m^2^ in Bhadrak, and 600−800 m^2^ in Mayurbhanj. Rice variety Swarna Sub-1 in Bhadrak and Swarna in Mayurbhanj were sown at the seed rate of 45−50 kg ha^−1^ using seed-cum-fertilizer drill. All other management practices were as per farmer’s practices. The fertilizer rates were same for all five treatments (Table 2). Fertilizer management and tillage operations were as per the drill-DSR treatment explained in Experiment I (see Section 2.2.1).

#### 2.2.3 Experiment III: evaluating seed rate and weed management in precision broadcast-DSR

The experiment was conducted in farmers’ fields in Mayurbhanj, Puri, and Bhadrak districts under rainfed conditions. In Mayurbhanj, the experiment was conducted for three years (2016–2018) in five to eight farmers’ fields, giving a total of 19 site-years. In Bhadrak it was replicated at five farmers’ fields only in 2017, while in Puri it was replicated in three farmers’ fields in 2016 and two fields in 2017. The size of the experimental plots was 400 m^2^ in Bhadrak and Puri, and 500−700 m^2^ in Mayurbhanj. Treatments included:

T1: Farmers’ practice of *beushening* (check): broadcasting high seed rate (100 kg ha^−1^) *fb* weed control by *beushening* operations at 30–35 DAS;

T2: Farmers’ practice of broadcasting the seeds at the rate of 100 kg ha^−1^, weed control by IWM program 1 (pretilachlor with safener @ 500 g *ai* ha^−1^ as PRE, *fb* bispyribac-sodium @ 20 g *ai* ha^−1^ at 15–25 DAS as POST, and *fb* one hand weeding at 30–35 DAS);

T3: Careful manual precision broadcasting of seeds at the rate of 60 kg ha^−1^, weed control by IWM program 1 (as in T2);

T4: Careful manual precision broadcasting of seeds at the rate of 60 kg ha^−1^, weed control by IWM program 2 (tank mix of bispyribac-sodium @ 20 g *ai* ha^−1^ + pyrazosulfuron-ethyl @ 20 g *ai* ha^−1^ at 15–25 DAS as POST *fb* one hand weeding at 30–35 DAS).

Rice varieties Swarna, Swarna Sub-1, and CR 1009 Sub-1 were broadcasted in Puri, Bhadrak and Mayurbhanj districts, respectively. In T1, *beushening* was practiced by wet ploughing and laddering at 30–40 DAS, and then rice seedling redistribution was done to have desired plant population as per farmers’ practices. Tillage operations before sowing for all treatments were similar to Experiment I, and all other management practices including NPK fertilizer applications were farmer’s practices. The fertilizer rates were similar in all treatments but varied by district (see Table 2). The fertilizer application timings were for T1 and T2-T4 similar to *beushening* and drill-DSR treatments, respectively, in Experiment I (see Section 2.2.1).

PRE and POST herbicides were sprayed using a knapsack sprayer with multiple nozzle boom (four nozzles) fitted with flat fan nozzle tips with a spray volume of 750 l ha^−1^ for PRE and 500 l ha^-1^ for POST application. PRE herbicides were sprayed 0–3 DAS while POST herbicides were sprayed as per treatments. Care was taken that there was no standing water in the field at the time of spraying.

### 2.3 Measurements and data analysis

Paddy was harvested at physiological maturity from three randomly selected 4.0 m^2^ areas in each treatment. Harvested paddy was threshed manually, grain moisture content was determined with a moisture meter, and grain yield was expressed at 14 % moisture content. The variable cost was calculated by summing the costs of seed, bullocks and tractor for field preparation, seed drill, fertilizers, herbicides, insecticides, and human labor. Unit costs for various inputs are given in Table 3. The gross return was calculated as the product of grain yield and farm gate price of paddy or un-milled rice (0.22 US$ kg^−1^). Net benefit for each treatment were calculated by deducting the variable cost from gross return. Benefit-cost ratio (BCR) was computed by dividing gross return with variable cost incurred in each treatment across different experiments.

**Table 3:** Unit cost of inputs for the calculation of variable costs and price of grain for calculating gross returns.

Inputs	Unit	Unit cost (US$)
**A. Total variable cost**		
1.Seed	kg^−1^	0.4
2.Establishment		
Bullocks for land preparation	8 h^−1^	11.3
Tractor for land preparation	h^−1^	8.5
Mini tiller	h^−1^	4.2
Seed drill	h^−1^	10
3.Fertilizers		
Urea	50 kg bag	4.5
SSP	50 kg bag	6.0
MOP	50 kg bag	7.5
DAP	50 kg bag	16.0
**4. Herbicides and insecticides**		
Pretiliachlor	l^−1^	8.5
Bispyribac-sodium	l^−1^	77.0
Pyrazosulfuron- ethyl	kg^−1^	35.2
motorized paddy weeder	h^−1^	7.0
Insecticides	l^−1^	11.3
5. Labor cost	8 h^−1^	2.8
**B. Gross return**		
Grain	kg^−1^	0.22

Exchange rate for 1 US$ equal to 71 INR.

In the R programming environment (version 3.6.1), using lme function we fitted the linear mixed-effect model for the dependent variables (e.g. yield, inputs, labor, cost, and net benefit) with treatments, districts and years in Experiment I and II, and treatments and combinations of district-years in experiment III, as fixed effects and farmers as a random effect. Post-hoc analysis (LSD-test) was used at P ≤ 0.05 to compare the differences among treatment means. A summary of statistical analysis for each experiment is presented in supplementary data (Supplementary Table 1–3).

## 3 Results

### 3.1 Experiment I: assessing performance of drill-DSR compared to beushening

All the interaction effects (treatment x district, treatment x year, treatment x district x year) were significant for labor use, crop establishment cost, weed control cost, variable cost, and share of weed control cost to variable costs (Supplementary Table 1). Therefore, treatment means for these variables are presented for all district and year combinations (Table 4). The labor use in weeding and/or *beushening* was lower in drill-DSR as compared to *beushening* in all years and districts, with the highest saving in Mayurbhanj (65–67 person-day ha^−1^) followed by Cuttack and Bhadrak (40–45 person-day ha^−1^). Irrespective of treatments, labor use was highest in Mayurbhanj (48–52 person-day ha^−1^) followed by Cuttack (37 person-days ha^−1^), and it was the lowest in Bhadrak (25–28 person-days ha^−1^). The seed rate was fixed for both *beushening* (100 kg ha^−1^) and drill-DSR (45 kg ha^−1^) for all districts and years; hence, the cost of seed was 22 US$ ha^−1^ lower in drill-DSR than in *beushening*. However, crop establishment cost was 7–15 US$ ha^−1^ higher with drill-DSR than *beushening* in all districts and years due to the hiring of seed drills. Weeds were managed by the integration of herbicides with hand weeding in drill-DSR; thus, overall weed management costs, including labor and herbicides, were 86–156 US$ ha^-1^ lower across the three districts in comparison to *beushening*. Variable costs were therefore lower in drill-DSR than *beushening* by 87–111, 159–171, and 108 US$ ha^−1^ in Bhadrak, Mayurbhanj, and Cuttack, respectively across the years. Herbicide-based IWM in drill-DSR resulted in a reduction in the share of weed control cost to variable cost by 17–21% across the three sites.

**Table 4:** Labor use, costs of establishment and weed management, variable cost and the share of weed management of variable cost in *beushening* and dry drill-DSR (Experiment I).

Variables*	Treatments	Bhadrak		Mayurbhanj		Cuttack
		2017	2018	2017	2018	2018
Labor for weeding/beushening (person-days ha^−1^)	*Beushening*	45	50	82	85	60
	Drill-DSR	5	6	15	20	15
	*LSD_0.05_*	*0*	*0*	*0*	*0*	*0*
Establishment cost (US$ ha^−1^)	*Beushening*	45	50	50	50	50
	Drill-DSR	60	60	57	57	57
	*LSD_0.05_*	*0*	*0*	*0*	*0*	*0*
Weed management cost (US$ ha^−1^)	*Beushening*	129	143	234	243	171
	Drill-DSR	43	47	78	98	71
	*LSD_0.05_*	*0*	*0*	*0*	*0*	*0*
Variable cost (US$ ha^−1^)	*Beushening*	441	474	568	576	444
	Drill-DSR	354	363	397	417	336
	*LSD_0.05_*	*18*	*11*	*0*	*0*	*1.3*
Share of weed management cost of variable cost (%)	*Beushening*	29	30	41	42	38
	Drill-DSR	12	13	20	24	21
	*LSD_0.05_*	*0.9*	*0.7*	*0.0*	*0.0*	*0.1*

*All the variables were significantly different (LSD test at P ≤ 0.05) between beushening and drill-DSR in both years in all districts.

For grain yield, net benefit, and B: C ratio, all interactions except treatment x district interaction were non-significant (Supplementary Table 1); therefore, treatment means are presented for each district (Table 5). Grain yield in drill-DSR as compared to *beushening* was 1.7 t ha^−1^ higher in Mayurbhanj, and 1.3 t ha^-1^ higher in Cuttack, but treatments did not differ statistically in Bhadrak (Table 5). However, the net benefit and BCR were significantly higher in drill-DSR compared to *beushening* in all the three districts due to the combination of increased yield and/or lower variable cost in drill-DSR. The largest net benefit was in Mayurbhanj (550 US$ ha^−1^) and the biggest BCR in Cuttack (4.8).

**Table 5:** Grain yield, net benefit and benefit cost ratio of *beushening* and drill-DSR (Experiment I).

Variables*	Treatments	Bhadrak (N = 10)	Mayurbhanj (N = 10)	Cuttack (N = 4)
Grain yield (t ha^−1^)	*Beushening*	5.14	3.30	5.97
	Drill-DSR	5.44	5.04	7.27
	*LSD_0.05_*	*0.43*	*0.14*	*1.10*
Net benefit (US$ ha^−1^)	*Beushening*	680	158	878
	Drill-DSR	846	708	1273
	*LSD_0.05_*	*95*	*35*	*293*
Benefit cost ratio	*Beushening*	2.5	1.3	3.0
	Drill-DSR	3.4	2.7	4.8
	*LSD_0.05_*	*0.3*	*0.9*	*0.8*

* All the variables were significantly different (LSD test at P ≤ 0.05) between beushening and drill-DSR in all three districts except grain yield at Bhadrak.

### 3.2 Experiment II: optimizing weed management in drill-DSR

All the interaction effects (treatment x year, treatment x district, and treatment x district x year) were non-significant for labor use, labor cost, weed management costs, variable cost, and share of weed management cost (Supplementary Table 2); therefore, data for these variables were pooled over districts and year and presented in Table 6. All the variables decreased in the following order: T1 (hand weeding twice) > T2 (PRE *fb* hand weeding) > T4 (POST *fb* hand weeding) > T3 (POST only) > T5 (mechanical weeding only). The labor used for weed control in T1 was nearly 2–4.5 times higher than herbicide-based treatments (T2 – T4), and nine times more than mechanical weeding (T5). Labor use was also significantly higher with PRE f*b* hand weeding (T2) than POST *fb* hand weeding (T4) treatment. Similarly, savings in the cost of labor for weeding in herbicide-based treatments (T2-T4) and mechanical weeding (T5) were 57–100 and 114 US$ ha^−1^, respectively compared to hand-weeding based T1. Total weed management costs were 28, 71, 57, and 100 US$ ha^-1^ lower in T2, T3, T4, and T5, respectively compared to T1. The difference in variable cost among the treatments were mainly due to weed management and the share of weed control cost to the variable costs was reduced from 29 % in T1 (hand weeding twice) to 15–24 % where herbicide was used (T2, T3 and T4) and as low as 8 % with mechanical weeding (T5).

**Table 6:** Labor use, costs of labor and weed management, variable cost and the share of weed management cost of variable costs in different weed management practices in dry drill-DSR conducted at farmers’ fields (Experiment II).

Treatment	Labor use (person-days ha^−1^)		Labor cost (US$ ha^−1^)		Weed management cost (US$ ha^−1^)		Variable cost (US$ ha^−1^)		Share of weed management costs (%)	
T1: HW twice	45	a	128	a	128	a	445	a	29	a
T2: PRE + HW	25	b	71	b	100	b	416	b	24	b
T3: POST only	10	d	28	d	57	d	373	d	15	d
T4: POST + HW	15	c	43	c	71	c	387	c	18	c
T5: MW once	5	e	14	e	28	e	344	e	8	e
*LSD_0.05_*	*0.0*		*0.5*		*0.5*		*4.4*		*0.3*	

*Mean within a column followed by same letter are not statistically different at the P < 0.05 level according to LSD test.

HW = hand weeding; PRE = pre-emergence herbicide; POST = post-emergence herbicide; MW = mechanical (motorized) weeder.

For grain yield and net benefit, the treatment x district interaction was significant whereas other interactions (treatment x year, and treatment x district x year) were non-significant (Supplementary Table 2); therefore, treatment means are presented for each district in Table 7. The weed management treatments had different effects on yield in the two districts, with no yield differences in Bhadrak but significant differences in Mayurbhanj (Table 7). In Mayurbhanj, grain yield was highest in T2 (PRE *fb* one hand weeding), which was on par with T1 (hand weeding twice). The grain yield in T4 (POST *fb* one hand weeding) was 0.3 t ha^−1^ lower than T2 (PRE *fb* one hand weeding) but similar to T1. The lowest yields were found in the treatments with no hand weeding (T3-POST only, and T5-mechanical weeding). For example, one hand weeding after POST (T4) resulted in a yield gain of 1.58 t ha^−1^ compared to POST only (T3).

**Table 7:** Grain yield and net benefit under different weed management practices in drill-DSR conducted at farmers’ fields in Bhadrak and Mayurbhanj (Experiment II).

	Grain yield (t ha^−1^)		Net benefit (US$ ha^−1^)	
Treatments	Bhadrak	Mayurbhanj	Bhadrak	Mayurbhanj
T1: HW twice	5.14 a	5.00 ab	695 c	661 b
T2: PRE + HW	5.28 a	5.21 a	756 b	735 a
T3: POST only	5.14 a	3.32 c	834 a	359 d
T4: POST + HW	5.47 a	4.90 b	827 a	694 ab
T5: MW once	5.28 a	3.53 c	827 a	434 c
*LSD_0.05_*	*0.50*	*0.27*	*56*	*60*

*Mean within a column followed by same letter are not statistically different at the P < 0.05 level according to LSD test.

HW = hand weeding; PRE = pre-emergence herbicide; POST = post-emergence herbicide; MW = mechanical (motorized) weeder.

Net benefit was significantly influenced by weed management practices in both districts (Table 7). In Bhadrak, since there was no significant difference in grain yield among the treatments, the difference in net benefit among treatments was primarily because of differences in variable cost. The highest net benefit (827–834 US$ ha^−1^) was recorded in the treatments with no hand weeding (T3 & T5) and one hand weeding after POST (T4), while the lowest net benefit (695 US$ ha^−1^) was found in the treatment with two hand weedings (T1). In Mayurbhanj, where there was a significant difference in grain yield among the treatments, net benefit was influenced by both grain yield and variable cost. Net benefit was highest in the treatments with combination of herbicide and one hand weeding (T2: PRE *fb* hand weeding, and T4: POST *fb* hand weeding), followed by T1 (hand weeding twice). Net benefit was lowest when there was no hand-weeding (T3) and mechanical weeding (T5).

### 3.3 Experiment-III: evaluating seed rate and weed management in precision broadcast-DSR

In this study, for all variables, the treatment x district-year combination interaction was significant (Supplementary Table 3). Therefore, pooled data across years are presented in Table 8, Table 9. At all three districts labor use was highest with *beushening* (T1) and approximately two to ten times higher than in the herbicide-based IWM treatments (T2-T4). Labor use was similar among the herbicide-based IWM treatments (T2-T4) in Bhadrak and Puri, while this differed in Mayurbhanj with 7–14 person-days ha^−1^ less labor use in treatments with PRE *fb* POST *fb* one hand weeding (T2 and T3) than treatment with POST *fb* one hand weeding (T4). Weed management costs were therefore at least 52 US$ ha^−1^ lower in each district with precision broadcast-DSR with herbicide-based IWM than *beushening* (T1). The variable cost was highest in *beushening* (T1) in all three districts, between 434 and 475 US$ ha^−1^ (Table 8). Compared to *beushening*, lower seed rate and herbicide-based IWM practices (T3 and T4) reduced the variable cost by 75–95 US$ ha^−1^ in Mayurbhanj, 73–107 US$ ha^−1^ in Bhadrak, 66–72 US$ ha^−1^ in Puri.

**Table 8:** Labor use for weed control, and weed management and variable costs, in precision broadcast-DSR with a combination of different seed rate and weed management practices (Experiment III).

Treatment	Labor use (person- days ha^−1^)		Cost of weed management (US$ ha^−1^)		Variable cost (US$ ha^−1^)	
**Mayurbhanj**						
T1: SR100+*Beushening*	83	a	185	a	475	a
T2: SR100+PRE + POST+HW	37	c	118	c	410	b
T3: SR60+PRE + POST+HW	30	d	103	d	380	d
T4: SR60+POST+HW	44	b	123	b	400	c
*LSD_0.05_*	*2*		*4*		*4*	
**Bhadrak**						
T1: SR100+*Beushening*	51	a	142	a	434	a
T2: SR100+PRE + POST+HW	7	b	82	b	374	b
T3: SR60+PRE + POST+HW	8	b	84	b	361	b
T4: SR60+POST+HW	5	b	50	c	327	c
*LSD_0.05_*	*3*		*8*		*15*	
**Puri**						
T1: SR100+*Beushening*	53	a	150	a	445	a
T2: SR100+PRE + POST+HW	18	b	92	b	388	b
T3: SR60+PRE + POST+HW	18	b	92	b	373	b
T4: SR60+POST+HW	24	b	98	b	379	b
*LSD_0.05_*	*19*		*42*		*46*	

Means within a column for a variable followed by the same letter are not different using LSD test at P ≤ 0.05.

SR100 = seed rate at 100 kg ha^−1^; SR60= seed rate at 60 kg ha^−1^; PRE = pre-emergence herbicide; POST = post-emergence herbicide; HW = hand weeding.

**Table 9:** Grain yield and net benefit in precision broadcast-DSR with a combination of seed rate and weed management practices (Experiment III).

	Grain yield (t ha^−1^)			Net benefit (US$ ha^−1^)		
Treatments	Mayurbhanj (R = 19)	Puri (R = 5)	Bhadrak (R = 5)	Mayurbhanj (R = 19)	Puri (R = 5)	Bhadrak (R = 5)
T1: SR100+*Beushening*	3.49 c	3.12 c	4.97 b	214 d	156 c	652 b
T2: SR100+PRE + POST+HW	3.88 b	3.80 b	4.42 c	354 c	349 b	591 b
T3: SR60+PRE + POST+HW	4.78 a	4.42 a	5.26 ab	567 a	489 a	787 a
T4: SR60+POST+HW	4.05 b	4.28 a	5.60 a	401 b	449 a	894 a
*LSD_0.05_*	*0.26*	*0.4*	*0.54*	*43*	*94*	*125*

Means within a column for a variable followed by the same letter are not different using LSD test at P ≤ 0.05.

SR100 = seed rate at 100 kg ha^−1^; SR60= seed rate at 60 kg ha^−1^; PRE = pre-emergence herbicide; POST = post-emergence herbicide; HW = hand weeding.

In all three districts, grain yields were consistently higher in treatments with the lower seed rate (60 kg ha^−1^) combined with herbicide-based IWM (T3 & T4) compared to *beushening* (T1), except T3 which was on par with T1 in Bhadrak. By replacing *beushening* (T1) with herbicide-based weed control without changing seed rate (T2), the average yield gain in Mayurbhanj and Puri was 0.39 and 0.68 t ha^−1^. In Bhadrak results were reverse with 0.5 t ha^−1^ higher yield in T1 than in T2 (Table 9). When *beushening* (T1) was replaced with precision broadcast-DSR with lower seed rate and herbicide-based weed control, the yield gain was 1.3 t ha^-1^ both in Mayurbhanj and Puri in T3 (PRE *fb* POST *fb* hand weeding), and 0.56 to 1.16 t ha^−1^ in T4 (POST *fb* hand weeding). Yield gain in Bhadrak was 0.63 t ha^−1^ in T4 compared to T1.

Net benefits were consistently higher in precision broadcast-DSR with lower seed rate and herbicide-based IWM (T3 and T4) compared to *beushening* (T1) in all the three districts (Table 9). Except at Bhadrak, these treatments effectively doubled the net benefit compared to *beushening* (T1). The combination of lower seed rate and herbicide-based IWM treatment (T3: PRE *fb* POST *fb* hand weeding) increased net benefits by 353 US$ ha^−1^ in Mayurbhanj, 333 US$ ha^−1^ in Puri, and 135 US$ ha^−1^ in Bhadrak compared to *beushening* (T1). Similarly, the combination of lower seed rate and other herbicide-based IWM (T4: POST *fb* hand weeding) increased net benefits by 187, 293, and 242 US$ ha^−1^ in Mayurbhanj, Puri and Bhadrak, respectively, compared to *beushening* (T1). By replacing *beushening* (T1) with herbicide-based weed control without changing seed rate (T2), net benefit was increased by 140 and 196 US$ ha^−1^ in Mayurbhanj and Puri, respectively, but did not differ in Bhadrak

## 4 Discussion

DSR has been compared with puddled transplanted rice in many environments and cropping systems with varying results. To our knowledge, this is the first study which assesses dry-DSR options (drill-DSR and precision broadcast-DSR) as potential alternatives to the relatively low-yielding and labor-intensive practice of *beushening*, a system that occupies around 6.4 million ha of lowland rice environment in Eastern India. Our results suggest that drill-DSR out-yields *beushening* by an average of 1.5 t ha^−1^ in two out of three districts and increases net benefits by 166–550 US$ ha^−1^ in all three districts (Table 5). A prime reason for higher yields is timely and effective weed control achieved through herbicide-based IWM. In *beushening*, early weed competition is generally higher as weeds are not controlled for the first 30–40 days prior to the *beushening* operation. Another reason for higher yield in drill-DSR could be due to more efficient use of applied fertilizer as fertilizers were applied at the recommended time (at sowing, 25–30 DAS, and panicle initiation stage). In *beushening*, fertilizers are not applied prior to *beushening* operations, which can be further delayed by insufficient rainfall.

One possible reason for the absence of yield advantage of drill-DSR over *beushening* in Bhadrak (Table 5) could be because access to irrigation in Bhadrak is good (62 % rice area in Bhadrak has access to irrigation at least once during critical period, Table 1), allowing for timely *beushening* operations. Another reason could be a more favourable soil type and hydrology (i.e. poor drained soils) that favor longer stagnation of water and hence better weed control, higher nutrient availability, and crop growth. These results suggest that *beushening* can produce yield similar to drill-DSR in agro-ecologies where weed pressure is low and beushening operations can be carried out in a timely manner. This hypothesis on timeliness needs to be tested so that advisory messages and options for weed control can be tailored to weed pressure, the reliability of the monsoon and access to irrigation.

The reduction in variable cost in drill-DSR was attributed to saving in seed costs (20 US$ ha^−1^) and weed control costs (114 US$ ha^−1^) due to a dramatic reduction in labor requirement of 40–65 person-days. *Beushening* for weed control and redistribution of rice seedlings is highly labor-intensive. These results suggest that drill-DSR can also address the emerging challenge of rising labor scarcity and associated increased labor costs, which have more than doubled in the last 10 years (Sudhir-Yadav Kumar et al., 2017).

The success of drill-DSR depends on new weed management practices. Hand weeding twice was effective in managing weeds in drill-DSR and can be an option if labor is affordable and available on time, though rarely is this the case in the region. Therefore, to reduce weed control costs and to achieve timely and effective weed control, cost effective and labor efficient alternatives such as mechanical weeding and herbicide-based IWM options are needed. The performance of weed management treatments in terms of yield varied with district (Table 7), which could be due to differential weed pressure as a result of soil and hydrological conditions which influence the duration of stagnation/flooding of fields which in turn suppresses weeds. In Bhadrak, conditions were more favorable for longer flooding/stagnation of rainwater in rice fields because of lowland topography, high water holding capacity of soil, and reliable land and irrigation facilities. Mayurbhanj, in contrast, has upland laterite soils with low water holding capacity and less access to irrigation.

This study found that the integration of herbicides (PRE or tank-mix application of POST) with one hand weeding can save labor and is more profitable and productive than hand-weeding, herbicide, or mechanical weeding alone. Similar results have been reported by many researchers (Bajiya et al., 2016; Godara et al., 2016; Yadav et al., 2011, 2014). The results of the current research are also in agreement with previous reports of superior weed control in DSR with sequential application of PRE (pendimethalin) followed by POST (bispyribac-sodium) (Singh et al., 2016; Walia et al., 2008) over hand weeding.

Insufficient availability of seed drills poses a major bottleneck to the broad adoption of drill-DSR in Odisha. As a lower-cost alternative to *beushening*, we therefore evaluated precision broadcast-DSR (i.e. lower seed rate combined with different weed management options) as an intermediate technology for areas where seed drills are not available. Our study shows that a combination of lower seed rate and herbicide-based IWM in precision broadcast-DSR led to higher grain yield, reduced labor use and higher profits similar to those obtained with drill-DSR. The higher yield in precision broadcast-DSR compared to *beushening* is probably largely due to timely weed control achieved using herbicides.

High seed rates in *beushening* are used to suppress weed growth in the absence of other weed control options. Our results show that high seed rates are not needed where weeds can be effectively controlled by herbicides, as others have observed (e.g. Rao et al., 2007). While the savings in seed cost are modest, around 20 US$ ha^−1^, this does offset a proportion of the herbicide cost and as we have shown increase profitability. The lower seed rate and integrated weed management practices in precision broadcast-DSR also gave almost similar advantages as drill-DSR, and hence this can be viable alternative option to *beushening* till seed drills are widely available in the state.

## 5 Conclusions

This study identifies new entry points in the form of drill-DSR or precision broadcast-DSR with IWM as an alternative to *beushening* that may improve the performance of rice across the 6.4 million ha in Eastern India where *beushening* is practiced. Yield gains from drill-DSR or precision broadcast-DSR are expected in production ecologies similar to Mayurbhanj, Puri and Cuttack. However, savings in production cost and higher profitability are expected across the entire geography where *beushening* is practiced. In addition, herbicide-based IWM results are likely relevant across rainfed lowland environments in South Asia in overcoming weed management constraints in all dry directly sown rice systems, including those replacing transplanted rice with dry seeding on non-puddled soils.

## CRediT authorship contribution statement

**P. Panneerselvam:** Data curation, Formal analysis, Writing - original draft, Supervision. **Virender Kumar:** Conceptualization, Formal analysis, Funding acquisition, Writing - original draft, Writing - review & editing, Supervision, Visualization. **Narayan Chandra Banik:** Data curation, Writing - review & editing. **Vivek Kumar:** . **Nabakishore Parida:** Data curation, Writing - review & editing. **Iftikar Wasim:** Writing - review & editing. **Aurovinda Das:** Writing - review & editing. **Sanghamitra Pattnaik:** Writing - review & editing. **Pravat Kumar Roul:** Writing - review & editing. **Dilip Ranjan Sarangi:** Writing - review & editing. **Pardeep K. Sagwal:** Writing - review & editing. **Peter Craufurd:** Writing - original draft, Writing - review & editing, Visualization. **Balwinder-Singh:** Writing - review & editing. **Ashok Yadav:** Writing - original draft, Writing - review & editing, Supervision. **Ram K. Malik:** Conceptualization, Funding acquisition, Supervision. **Sudhanshu Singh:** Conceptualization, Funding acquisition, Writing - review & editing. **Andrew J. McDonald:** Conceptualization, Funding acquisition, Writing - review & editing, Visualization.

## Declaration of Competing Interest

The authors report no declarations of interest.
