# Supplementary material for: Transforming labor requirement, crop yield, and profitability with precision dry-direct seeding of rice and integrated weed management in Eastern India
Source: Field Crops Res. 2020 Dec 15;259:107961. doi: 10.1016/j.fcr.2020.107961 (PMC8188295; doi:10.1016/j.fcr.2020.107961)
Supplement: Supplementary file 1 [file mmc1.docx]

Supplementary:

Table 1. Statistical analysis for experiment I: Beushening and drill-DSR

|  | Labor days | Establishment cost | Weed mgt cost | Variable cost | Share of weed manage costs | Grain yield | Net benefit | BCR |
| --- | --- | --- | --- | --- | --- | --- | --- | --- |
| Treatments (T) | *** | *** | *** | *** | *** | *** | *** | *** |
| District (D) | *** | *** | *** | *** | *** | *** | *** | *** |
| Year (Y) | *** | *** | *** | *** | *** | NS | NS | ** |
| T x D | *** | *** | *** | *** | *** | *** | *** | *** |
| T x Y | *** | *** | *** | NS | *** | NS | NS | NS |
| T x D x Y | *** | *** | *** | *** | *** | NS | NS | NS |

Significant codes: ‘***’ significant at 0.1 %; ‘**’ significant at 1%; ‘*’ significant at 5%;

NS means non-significant (p value is equal or more than 0.1)

While these tables show the actual level of significance, all LSDs are reported for p ≤ 0.05

Table 2. Statistical analysis for experiment II: Weed management practices in drill-DSR

|  | Labor (persons days ha^-1^) | Labor cost for weeding | Weed mgt cost | Variable cost | Share of weed manage costs | Grain yield | Net benefit |
| --- | --- | --- | --- | --- | --- | --- | --- |
| Treatments (T) | *** | *** | *** | *** | *** | *** | *** |
| District (D) | NS | NS | NS | *** | *** | *** | *** |
| Year (Y) | NS | NS | NS | ** | * | ** | *** |
| T x D | NS | NS | NS | NS | NS | *** | *** |
| T x Y | NS | NS | NS | NS | NS | NS | NS |
| T x D x Y | NS | NS | NS | NS | NS | NS | NS |

Significant codes: ‘***’ significant at 0.1 %; ‘**’ significant at 1%; ‘*’ significant at 5%;

NS means non-significant (p value is equal or more than 0.1)

While these tables show the actual level of significance, all LSDs are reported for p ≤ 0.05

Table 3. Statistical analysis for experiment III: Precision broadcast-DSR

|  | Labor for weeding | Cost of weed management | Variable cost | Yield | Net benefit |
| --- | --- | --- | --- | --- | --- |
| Treatments (T) | *** | *** | *** | *** | *** |
| District-Year (DY) | *** | *** | *** | *** | *** |
| T x DY | *** | ** | * | *** | *** |

Significant codes: ‘***’ significant at 0.1 %; ‘**’ significant at 1%; ‘*’ significant at 5%;

NS means non-significant (p value is equal or more than 0.1)

While these tables show the actual level of significance, all LSDs are reported for p ≤ 0.05
